# A Psychometric Evaluation of the Santa Clara Strength of Religious Faith Questionnaire among Students from Poland and Chile

**DOI:** 10.1007/s11089-017-0754-4

**Published:** 2017-02-03

**Authors:** Marcin Wnuk

**Affiliations:** Adam Mickiewicz University, Poznań, Poland

**Keywords:** Religiosity, Spirituality, Students, Chile, Poland

## Abstract

This study verifies the psychometric properties of the Santa Clara Strength of Religious Faith Questionnaire (SCSORFQ). This measure consists of 10 items regarding strength of religious faith, regardless of religious denomination or affiliation. Participants were 177 students from Chile and 393 students from Poland. The SCSORFQ method is a reliable measure with good internal consistency evaluated by the α-Cronbach coefficient. Factor analysis statistically confirmed the unidimensional structure of the SCSORFQ. The strength of religious faith in both student groups was moderate to strong correlated with public or private aspects of religiousness and strong correlated with spiritual experiences. It was weakly correlated with the cognitive indicator of well-being as satisfaction with life as well as with existential variables like hope and meaning of life. The results confirm that the SCSORFQ is psychometrically sound and is therefore recommended for use by researchers studying the construct of religiousness.

Within recent years, there has been a notable rise of interest in the role that religion and spirituality play in the quality of an individual’s life and well-being, and new tools for their study have been developed (Egbert et al. 2001; Francis 1993; Kilpatrick et al. 2005; Lewis et al. 2005; O’Connell and Skevington 2007; Plante et al. 2002; Storch et al. 2004b; Worthington et al. 2003). The literature on religiousness regards this concept as similar to spirituality but as either a broader construct (Hill et al. 2000; Pargament 1999) or a narrower construct (Cawley 1997; Nagai-Jacobson and Burkhardt 1989; Sawatzky et al. 2005).

Many recent studies have emphasised the multifaceted character of both concepts (LaPierre 1994; Wortmann and Park 2008) in that some aspects of religiousness are closely related to spirituality but others much less so (Heintz and Baruss 2001). Researchers have a tendency to focus on specific aspects of religiousness connected with personal experience and practices such as prayer (Fisher et al. 2002; Francis and Kalder 2002), reading the Bible (Ayele et al. 1999; Francis 2000; Francis and Evans 1996), attending church (Demir and Urberg 2004; Lewis 2002; Steinitz 1980), or religious denomination (Abbotts et al. 2004; Ross 1990). Confronted with life’s daily problems, religion can be used to cope with stress (Pargament et al. 2000; Pargament et al. 2001; Pargament et al. 1998; Phillips et al. 2004). Religious coping is recognized as a multidimensional process that cannot be reduced to simple behavioural indicators (e.g., prayer, going to a place of worship) or restricted to passive or defensive functions of the psyche (denial, rationalization, etc.). It incorporates active, passive, problem-focused, emotion-focused, intrapsychic (i.e., cognitive, behavioural), and interpersonal methods of managing stress (Klaassen et al. 2006).

In order to determine if a positive association exists between health or well-being and a postulated sense of religious maturity, then the motivation for religiousness needs to be divided into internal and external forms (Allport 1966). The supposition that religious maturity has autotelic and non-instrumental characteristics that conflict with the quality of life is reflected in the literature. According to research results, internally motivated religiousness is positively related to health whereas externally motivated religiousness is either negatively correlated with or unrelated to health (Ventis 1995). According to Batson and Schoenrade (1991), the appearance of religious maturity that conflicts with health is the so-called ‘religion as quest’ orientation. Results of the study between this religious orientation with various indicators of health demonstrate equivocal findings and are in opposition to the conclusions made in another study (Ventis 1995).

The above controversies reflect a lack of reliable measures in the literature for studying religiousness that both have a universal character independent of cultural and belief contexts and also are simple and easy to use and reflect various religious-spiritual aspects. It seems, however, that the Santa Clara Strength of Religious Faith Questionnaire (SCSORFQ) fulfils these requirements. Initially, the SCSORFQ was used in clinical studies with patients suffering from a chronic illness because of its short and simple construction (Plante et al. 2000; Plante et al. 1999; Sherman et al. 1999). This measure has found implementation in other populations (Edwards et al. 2002; Freiheit et al. 2006; Plante and Boccaccini 1997a; Plante et al. 2000; Plante et al. 1999). The main advantage of this procedure lies in its universal applicability. For example, it is independent of denomination or ethnic background of the participants (Sherman et al. 1999).

The SCSORFQ has good psychometric properties (Plante and Boccaccini 1997a, b; Sherman et al. 2001). Many studies indicate that SCSORFQ has a good internal consistency. This measure consists of one factor (Freiheit et al. 2006; Lewis et al. 2001; Wnuk 2009a). Reliability of SCSORFQ measuring the α-Cronbach coefficient ranges from 0.93 to 0.97, depending on the population (Lewis et al. 2001; Sherman et al. 1999; Sherman et al. 2001; Strawser et al. 2004). Reliability of the SCSORFQ using the test-retest method, with a 3 week delay, gave values of α ranging from 0.93 in patients with breast cancer to 0.83 in healthy adults (Sherman et al. 2001).

The theoretical consistency of the SCSORFQ was verified by relating its results with other indicators of religiousness. Depending on those indicators, the strength of the relationship was high or was borderline between moderate and high.

The SCSORFQ was correlated with intrinsic religiousness amongst gynecological patients (Sherman et al. 1999), students (Plante et al. 1999; Wnuk 2009a), alcohol-dependent persons (Plante et al. 1999; Wnuk 2009a), and healthy adults (Sherman et al. 2001). Strength of religious faith was related to private or public forms of religiousness (Sherman et al. 1999) as well as positive religious coping (Freiheit et al. 2006; Sherman et al. 1999; Wnuk 2009a).

The SCSORFQ was positively related to meaning of life among students, gynecological patients, and patients with bone marrow cancer (Plante et al. 2000; Sherman et al. 1999), moderately related to forgiveness in a sample of married Catholic couples (Batson et. al. 2006), and positively correlated with forgiveness and hope amongst students (Edwards et al. 2002; Plante and Boccaccini 1997a). In a sample of students, there was no relationship between the strength of religious faith and positive or negative affect (Freiheit et al. 2006) or depression (Plante et al. 1999). Furthermore, in five student groups, a link to fear was found only in one (Plante et al. 1999; 2000).

The aim of this study was to verify if SCSORFQ has good psychometric properties regarding reliability using the α-Cronbach method, internal consistency using factor analysis, and theoretical consistency using the r-Pearson coefficient with othe measures.

## Method

### Participants

#### Sample 1

Sample 1 consisted of university students from Chile, *n* = 177. In the study sample, 62% participants were men and 38% were women. Questionnaires were given to students after classes and, when ready, collected by a Polish-grant-funded overseas student. The average age of the Chilean students was 21.35 years (SD = 1.8), and all participants had secondary education. Those professing the Roman Catholic faith were 55.37% of the participants, and the rest were Evangelicals, Mormons, Protestants, Seventh-day Adventists (23.16%) or considered themselves either agnostic, atheistic, or non-believing (21.47%).

#### Sample 2

The students in sample 2, *n* = 393, aged 22.9 years (SD = 2.8), were attending the Bydgoszcz (Bromberg) School of Higher Education in Poland in the 2011/2012 semester and were studying physiotherapy, rehabilitation, or public health. Questionnaires were handed out and filled in during a work day. Women were 84.7% of the participants and men 15.3%. They represented the following religious denominations: Roman Catholics 93.1%, Buddhists 1.3%, and 5.6% other traditions. 91.1% of the students had a secondary education and 8.9% had higher education.

### Measures

Every measure that was used was translated into the participants’ native languages. Additionally, the SCSORFQ was back-translated into English in the process of obtaining translation equivalency. Both samples in this study were presented most of the measures, as described below, although one sample may have received a few that the other did not. The following research tools were used.

The Daily Spiritual Experiences Scale (DSES) consists of 16 questions, each one having 6 grades ranging from 1 (*never or almost never*) to 6 (*many times daily*). The more points scored, the greater the person’s spirituality. The scale’s reliability, depending on population, ranges from α = 0.86 to 0.95 (Loustalot et al. 2006; Wnuk 2009b). The short version of this measure was used, which consists of six items.

The Brief Religious Coping Scale (Brief RCOPE) is comprised of 14 items regarding positive and negative religious coping. Each question has a 4-point graded scale depending on how much the subject agrees/disagrees with the question. The more points scored, the more frequently the individual uses religious coping. The scale’s reliability, depending on population, ranges from α = 0.78 to 0.94 (Pargament et al. 2000). In the study, only the items regarding positive religious coping were asked. An additional measure of religiousness was based on 5-point scale for how often a given subject attended Mass. This consisted of (1) *never with the exception of baptisms, marriages, or funerals*, (2) *a few times a year*, (3) *1–2 times monthly*, (4) *2–3 times monthly*, and (5) *once per week or more*. The scale for measuring how often the participants prayed consisted of *never*, *sometimes*, *once monthly*, *once weekly*, and *every day*.

The Positive and Negative Affects Schedule (PANAS) consists of 10 statements related to positive emotional states and another 10 concerning negative ones. Each question is graded from 1 = *a little or none* up to 5 = *very frequently*. The more points scored, the greater a person’s negative as well as positive affect. Participants were asked about their assessments of their emotional state based on how often they felt in response to particular questions up to the weekend before being surveyed. The reliability scale varied, according to studies, from α = 0.86 to 0.89 for the positive affect and α = 0.84 to 0.85 for the negative affect (Crawford and Henry 2004; Trawka and Derbis 2006). Among students, the reliability of this measure using the test-retest method ranged from 0.39 to 0.71 (Trawka and Derbis 2006).

The Satisfaction with Life Scale (SWLS) is a universally used tool to measure mental well-being based on an operational concept of satisfaction with life defined through a conscious assessment/judgement of one’s life through a comparison to self-imposed standards (Diener et al. 1985). This measure consists of five statements to which each individual gives an answer according to a graded 7-point scale; the greater the points, the more satisfaction there is with life, according to this measure. This scale possesses satisfactory psychometric properties. Its reliability is 0.83 as determined by the test-retest method after a 2-week repeated study, which rose to 0.84 after a month but then ranged between 0.64–0.82 after 2 months (Pavot and Diener 1993). The more points scored, the greater a person’s life satisfaction. The method’s unequivocal nature has been confirmed by various studies (Diener et al. 1985; Lewis et al. 1995; Pavot et al. 1991; Shevlin and Bunting 1994).

The level of stress was evaluated by six items concerned with negative feelings such as being irritated and feelings of worthlessness, hopelessness, and sadness, of which the latter two had the options of *never*, *rarely*, *normally*, *frequently*, and *always*. The more points, the higher level of stress the participant was feeling. This measure has been adopted by the European Centre for Disease Prevention and Control (ECDC) in dealing with illness (http://www.cdc.gov/nchs/nhis.htm). The reliability of the scale in measuring the α-Cronbach coefficient was 0.82.

The Purpose in Life Test (PIL) consists of 20 questions on the meaning of life, each one graded from to 1 to 7, where 7 denotes a maximal sense for having a meaning of life and 1 is the lowest. Results are calculated by the sum totals for all questions. The higher the score, the greater one’s sense of one’s meaning of life/purpose being fulfilled, whereas the lower the score, the larger the frustration with one’s existence. The range of possible results so obtained lies between 20 and 140 points (Cekiera 1985). The reliability of this method is 0.9 (Crumbaugh and Maholic 1964). When a Polish version of this scale was used for the test-retest method, after 6 months these reliability values ranged from 0.64 to 0.7 depending on the population studied (Siek 1993).

The Transgression-Related Interpersonal Motivations Scale (TRIM-12) is a motivational measure of forgiveness (McCullough et al. 1998). This measure comprises 12 questions, of which five are related to revenge/vengefulness whereas seven regard avoidance behaviour towards the transgressor. The more points scored, the greater a person’s forgiveness indicator. Each question is rated on a 5-point Likert scale ranging from 1 = *categorically disagree* to 5 = *decidedly agree*. Results are then based on the score totals for all answers given. Reliabilities of relative stability (Cronbach’s α) for the revenge and avoidance subscales were respectively 0.9 and 0.86–0.94, and test-retest reliability was likewise respectively 0.53–0.79 and 0.44–0.86 (with a 3–9 week deferral). This scale shows high internal consistency using factor analysis as well as theoretical consistency obtained through correlation and other measures of forgiveness and similar constructs (McCullough et al. 1998).

The Herth Hope Index (HHI) is a 12-question measure on a 4-point scale ranging from 1 = *categorically agree* to 4 = *definitely disagree* (Herth 1992). The more points scored, the greater the person’s hope. This scale has properties advantageous to psychometry; the scale’s reliability in a population suffering from illness is α = 0.97 and 0.91 using the test-restest method (Herth 1992).

### Procedures

Participants were consenting students who answered a survey designed to study their psychosocial well-being and religiousness. This consisted of a 1-h questionnaire that students took home to complete and return during the next day’s class. Data analysis was performed by the SPSS statistical package. The internal consistency was determined by factor analysis, whereas theoretical consistency was verified using paired correlation. The reliability was calculated by the Cronbach α coefficient. Tables 1 and 2 show the descriptive statistics for Chilean and Polish students, respectively.

**Table 1:** Descriptive statistics and reliabilities of scales for the chilean student group

Measure	Chilean students (*n* = 177)		
	Mean	SD	Reliability
SCSORFQ	28.67	11.39	0.95
Frequency of praying	2.84	1.47	
Frequency of attending mass	2.81	1.90	
DSES	18.22	6.74	0.85
PANAS			
Positive affect Negative affect	16.47 14.06	3.51 4.22	0.72 0.82
SWLS	27.04	5.41	0.83
Stress level	13.85	7.32	0.82
HHI	36.76	4.62	0.77

**Table 2:** Descriptive statistics and reliabilities of scales for polish student group

Measures	Polish students (*n* = 393)		
	Mean	SD	Reliability
SCSORFQ	34.93	8.77	0.93
Frequency of praying	3.16	1.49	-
Frequency of attending mass	3.24	1.51	-
DSES	18.61	5.53	0.86
RCOPE			
Positive religious coping	18.01	6.24	0.76
HHI	37.56	4.25	0.76
PIL	104.62	17.66	0.78
TRIM	30.09	5.87	0.88
SWLS	28.31	5.12	0.74
Gender			
Male Female	35.68 34.98	7.93 8.93	

## Results

### Reliability

The reliability of the SCSORFQ measuring the α-Cronbach coefficient was 0.952 for the Chilean students and 0.933 for the Polish students. Reliabilities of all other scales in the study are reported in Tables 1 and 2.

### Theoretical consistency

The correlation coefficients are presented in Table 3. In both samples, there is a strong positive correlation between the strength of religious faith and spiritual experiences. A moderately strong correlation was observed between frequency of prayer and frequency in attending Mass. Among Polish students, the strength of religious faith was also moderately and positively associated with positive religious coping. For non-religion-affiliated Chilean students, the strength of their religious faith was related to frequency of prayer and was not correlated with frequency of attending Mass, life satisfaction, or hope (see Table 3). In the group of religion-affiliated Chilean students, the strength of religious faith was positively correlated with hope, life satisfaction, and frequency of attending Mass (see Table 3).

**Table 3:** Correlation between SCSORFQ with other scales amongst polish and Chilean students

	Polish students (*n* = 393)	Chilean students regardless of religious affiliation (*n* = 177)	Chilean students with religious affiliation (*n* = 141)	Chilean students without religious affiliation (*n* = 36)
Frequency of praying	0.61**	0.67**	0.62**	0.36*
Frequency of attending Mass	0.64**	0.63**	0.61**	−0.04
Frequency of spiritual experiences	0.77**	0.75**	0.70**	0.59**
Hope	0.14*	0.17*	0.27*	0.28
Satisfaction with life	0.12*	0.23*	0.26**	0.26
Positive religious coping	0.62**	-	-	-
Purpose of life	0.16*	-	-	-
Forgiveness	0.34**	-	-	-
Positive affect	-	0.13	0.05	0.29
Negative effect	-	−0.01	−0.05	−0.07
Stress level	-	0.11	0.08	−0.02

*p ≤ 0.05

**p ≤ 0,01

- Means that the scale was not administered to the sample

In the sample of Chilean students, there was no relationship between strength of religious faith and the level of stress and negative as well as positive affect, regardless of religious affiliation. Among the Polish students, the strength of religious faith was weakly related to life satisfaction, hope, and the meaning of life. Furthermore, the strength of religious faith was positively and moderately correlated with forgiveness. Women from both Poland and Chile had similar levels of religious faith compared to men: for Polish women, *t* = 1.41, *p* = 0.159 (M = 34.78; SD = 8.93 versus M = 35.68; SD = 7. 93), and for Chilean women, *t* = −0.78, *p* = 0.433 (M = 29.61; SD = 11.62 versus M = 27.14; SD = 11.00).

### Internal consistency

For the Chilean students, the adequacy of the sampling was measured by an index value obtained from the Kaiser-Meyer-Olkin (KMO) test, indicating the proportion of the total variance that can be explained by the observed index value of 0.932; this showed a highly satisfactory sampling. Bartlett’s test of sphericity was also used to show the significance of correlation, if any, amongst the chosen variables; this demonstrated a Chi^2^ value =1538.395, DF = 45, and significance of *p* < 0.05, thus again indicating adequate sampling (see Table 4). The Principal Components Analysis method used for factor analysis confirmed that the SCSORFQ is a one-factor measure (i.e., strength of religious faith), which accounts for 69.95% of the variance of this measure.

**Table 4:** Rotated component matrix

	Component	Component
SCSORF items	Chilean students	Polish students
1	0.85	0.80
2	0.73	0.69
3	0.88	0.81
4	0.87	0.84
5	0.80	0.76
6	0.88	0.86
7	0.85	0.90
8	0.79	0.67
9	0.80	0.83
10	0.87	0.81

In similar fashion (see Table 4), Polish students tested by the KMO method yielded an index value of 0.921, thus also confirming the adequacy of sampling. Furthermore, Bartlett’s test of sphericity showed more significant correlations between the aforementioned variables, (Chi^2^ value =2355.087, DF = 45, and significance of *p* < 0.01). As previously, the Principal Components Analysis method used for factor analysis again confirmed that the SCSORFQ is an instrument composed of one factor (i.e., strength of religious faith), which accounts for 64.25% of the variance of this measure.

## Conclusions

The aim of this study was to verify the psychometric properties of the SCSORFQ amongst samples of Polish and Chilean students. In accordance with recent research, the satisfactory internal consistency and reliability of this measure has been confirmed (Lewis et al. 2001; Plante and Boccaccini 1997a, 1997b; Storch et al. 2004a; Wnuk 2009a). Consistent with other studies, the results confirmed the single-factor structure of this measure (strength of religious faith) for both the Polish and Chilean samples (Lewis et al. 2001; Plante and Boccaccini 1997a, 1997b; Storch et al. 2004a; Wnuk 2009a). Correlations of this factor with other questionnaire items ranged from 0.73 to 0.88 and 0.67 to 0.86 for Polish and Chilean students respectively, demonstrating that each of the statements are good indicators of the strength of religious faith in both populations. Among Chilean students, the unidimensional construct accounted for a higher amount of the variance than in the Polish sample—69% versus 64.25%—thereby showing a greater internal consistency in the former compared to the latter. Recent research conducted among students from different countries also confirmed a satisfactory level of reliability of the SCSORFQ (Freiheit et al. 2006; Lewis et al. 2001; Plante and Canchola 2004; Plante et al. 2001; Plante et al. 2000). In the Polish sample, the α-Cronbach coefficient was α = 0.952, and in the Chilean sample it was 0.933.

This study confirmed the theoretical consistency in both samples. Other studies (Freiheit et al. 2006; Plante et al. 1999 **;** Sherman et al. 1999; Sherman et al. 2001) have also shown that the SCSORFQ results are strongly or moderately related to measures of religiousness. For example, in both student groups, the strength of religious faith was strongly correlated with spiritual experiences and was borderline moderately to strongly correlated with public and private aspects of religiousness. In the sample of Polish students, there was a positive relationship between the SCSORFQ results and religious coping (Freiheit et al. 2006).

Among the Chilean students, independence of religious affiliation strength of religious faith were not related to positive or negative affect or to level of stress (Freiheit et al. 2006). In a sample of Polish students as well as religion-affiliated Chilean students, there were only weak correlations between strength of religious faith and satisfaction with life. Based on previous studies of students, it can be said that the strength of religious faith is a correlative indicator, both positive and cognitive, of well-being, examples being satisfaction with life when there are no links with either negative or affective measures. According to previous studies, strength of religious faith correlated weakly and positively with hope (Plante and Boccaccini 1997a) and meaning of life (Plante et al. 2000) as well as moderately with forgiveness (Edwards et al. 2002).

This study confirms that the SCSORFQ is a valuable and reliable measure that can be used successfully on Polish and Chilean students. According to the results, the SCSORFQ is more reliable and internally consistent for Chilean than Polish students, but nevertheless, the latter sample still demonstrated excellent psychometric properties. Although participants in both samples had similar ages and levels of education, they had different men-to-women ratios and different patterns of denominations and religious affiliations. The sample of Polish students had a higher proportion of women, and almost all the research participants were members of the Roman Catholic Church. In contrast, the Chilean students showed a wider range of outlooks on life, including agnosticism and atheism, therefore showing that the instrument can also be used to study the religiously skeptical. In both samples, the SCSORFQ results were independent of gender. Among non-religiously affiliated Chilean students, the strength of religious faith was related to frequency of prayer and not correlated with frequency of attending Mass. This means that among the Chilean students, using prayer is characteristic only of individuals with strong faith. This requires some explanation. Probably those students have religious roots and practise prayer to help them cope with daily stress and difficult situations. At the same time, their strength of faith is independent of their attendance at Mass and is coherent with their worldview. Religious affiliation was a factor moderating the relationship between religious faith and hope and between religious faith and life satisfaction as well as religious faith and frequency of attending Mass (see Table 3). For the students who had a religious affiliation, strength of religious faith was related to frequency of attending Mass, life satisfaction, and hope. In the group of students with no religious affiliation, religious faith did not correlate with frequency of attending Mass, life satisfaction, or hope. They probably have non-religious and secular sources of hope and life satisfaction. Interestingly, among the students who were not religiously affiliated, strength of religious faith was positively correlated with spiritual experiences. This means that spiritual experiences are not related to religious background alone; it is testimony that non-religious Chilean students can have spiritual experiences.

The results of this study support the universal adoption of the SCSORFQ for studies of students irrespective of their age, gender, cultural background, ethnicity, or denomination. The SCSORFQ can be effectively used to determine how active the role of religion and spiritual experiences is among Polish and Chilean students, notwithstanding whether religiousness can be treated in terms of defining one’s identity or whether it is considered to be a more narrow or a broader concept than spirituality.
